# The Appearance Order of Varying Intervals Introduces Extra Modulation Effects on Neuronal Firing Through Non-linear Dynamics of Sodium Channels During High-Frequency Stimulations

**DOI:** 10.3389/fnins.2020.00397

**Published:** 2020-05-19

**Authors:** Lvpiao Zheng, Zhouyan Feng, Hanhan Hu, Zhaoxiang Wang, Yue Yuan, Xuefeng Wei

**Affiliations:** ^1^Key Laboratory of Biomedical Engineering for Ministry of Education, College of Biomedical Engineering and Instrument Science, Zhejiang University, Hangzhou, China; ^2^Department of Biomedical Engineering, The College of New Jersey, Ewing, NJ, United States

**Keywords:** high-frequency stimulation, inter-pulse interval, *in vivo* experiment, computational modeling, hippocampal pyramidal cell, membrane dynamics, non-linearity, population spike

## Abstract

Electrical pulse stimulation in the brain has shown success in treating several brain disorders with constant pulse frequency or constant inter-pulse interval (IPI). Varying IPI may offer a variety of novel stimulation paradigms and may extend the clinical applications. However, a lack of understanding of neuronal responses to varying IPI limits its informed applications. In this study, to investigate the effects of varying IPI, we performed both rat experiments and computational modeling by applying high-frequency stimulation (HFS) to efferent axon fibers of hippocampal pyramidal cells. Antidromically evoked population spikes (PSs) were used to evaluate the neuronal responses to pulse stimulations with different IPI patterns including constant IPI, gradually varying IPI, and randomly varying IPI. All the varying IPI sequences were uniformly distributed in the same interval range of 10 to 5 ms (i.e., 100 to 200 Hz). The experimental results showed that the mean correlation coefficient of PS amplitudes to the lengths of preceding IPI during HFS with random IPI (0.72 ± 0.04, *n* = 7 rats) was significantly smaller than the corresponding correlation coefficient during HFS with gradual IPI (0.92 ± 0.03, *n* = 7 rats, *P* < 0.001, *t*-test). The PS amplitudes induced by the random IPI covered a wider range, over twice as much as that induced by the gradual IPI, indicating additional effects induced by merely changing the appearance order of IPI. The computational modeling reproduced these experimental results and provided insights into these modulatory effects through the mechanism of non-linear dynamics of sodium channels and potassium accumulation in the narrow peri-axonal space. The simulation results showed that the HFS-induced increase of extracellular potassium ([K^+^]_*o*_) elevated the membrane potential of axons, delayed the recovery course of sodium channels that were repeatedly activated and inactivated during HFS, and resulted in intermittent neuronal firing. Because of non-linear membrane dynamics, random IPI recruited more neurons to fire together following specific sub-sequences of pulses than gradual IPI, thereby widening the range of PS amplitudes. In conclusion, the study demonstrated novel HFS effects of neuronal modulation induced by merely changing the appearance order of the same group of IPI of pulses, which may inform the development of new stimulation patterns to meet different demands for treating various brain diseases.

## Introduction

Deep brain stimulation (DBS) generally refers to utilizing sequences of electrical pulses to treat neurological and psychiatric disorders in the brain (Lozano and Lipsman, 2013). In theory, electrical stimulations on the neuronal membrane could generate various modulation effects on neuronal activity to treat various brain diseases. However, current DBS therapy has only been popularized in treating a few diseases such as movement disorders and refractory epilepsy (Bergey, 2013; Wichmann and DeLong, 2016; Cury et al., 2017). Besides the lack of understanding of the exact pathophysiological mechanisms of many brain diseases, extending DBS treatment to other diseases is limited by a lack of versatility in stimulation paradigms, as well as a lack of understanding of various stimulation effects on neurons and their mechanisms.

Major parameters involved in the design of pulse stimulation paradigms include pulse amplitude, pulse width, and pulse repetition rate (frequency). In particular, the efficiency and efficacy of DBS therapy are strongly dependent on the pulse frequency [or inter-pulse interval (IPI)]. Common DBS therapy utilizes pulse sequences with a constant frequency of around 130 Hz (Cagnan et al., 2017; Dandekar et al., 2018), termed as high-frequency stimulation (HFS). For example, a frequency in 90–185 Hz was proven effective in suppressing tremor of Parkinson’s disease in clinic (Rizzone et al., 2001; Bittar et al., 2005; Dayal et al., 2017). Frequencies in a similar range have also been applied to control epileptic seizures (Cukiert et al., 2011; Fisher and Velasco, 2014; Laxpati et al., 2014).

Besides constant IPI, irregular temporal patterns of stimulation (i.e., sequences with varying IPI) have been exploited to improve the DBS therapies as well as to probe the underlying mechanisms of DBS (Grill, 2018). For example, stimulations with random IPI have emerged as a strategy to suppress the epileptic activity of neural networks (Cota et al., 2009; Mesquita et al., 2011; Medeiros et al., 2012; Santos-Valencia et al., 2019) and to affect central nervous system arousal (Quinkert et al., 2010; Quinkert and Pfaff, 2012; Tabansky et al., 2014). These studies demonstrated a significant effect of temporally random IPI on behavioral outcomes of seizure suppression or CNS arousal evaluated on experimental animals. Also, stimulations with varying IPI of different patterns have been used to suppress tremor (Birdno et al., 2007; Kuncel et al., 2012; Swan et al., 2016). However, the effects of stimulations and mechanisms of action with varying IPI are uncertain yet, limiting the application and development of irregular HFS in clinic.

Previous studies have shown that HFS with constant IPI can only intermittently generate action potentials in neurons because the HFS-induced depolarization block on the neuronal membrane prevents the neurons from firing in response to each stimulation pulse (Jensen and Durand, 2009; Feng et al., 2013; Rosenbaum et al., 2014). Intermittent activation of individual neurons may generate asynchronous firing in neuronal populations (Popovych and Tass, 2014; Feng et al., 2017; Wang et al., 2018). Pulses with a higher frequency (i.e., a shorter IPI) cause a deeper depolarization block, allowing less neurons to be recruited to fire by every pulse, as indicated by smaller evoked population spikes (PSs) during steady-state period of HFS (Feng et al., 2013, 2014). However, a recent study has shown that even with higher instantaneous frequencies for all pulses, stimulations of varying IPI with a random order can occasionally recruit more, rather than less, neurons to fire simultaneously than stimulations with constant IPI (Feng et al., 2019). This suggests that the recruitment of neuronal firing could be affected not only by pulse frequencies but also by the temporal orders of varying IPI. However, the underlying mechanisms are not clear. The purpose of this study is to unveil the cellular mechanisms of neuronal responses to HFS with different temporal orders of varying IPI, which may promote the development of novel stimulation paradigms of DBS for treating more diseases.

Membrane dynamics of ionic channels, such as Na^+^ and K^+^ channels, change non-linearly with time and with transmembrane potential (Hodgkin and Huxley, 1952). We hypothesize that a random arrangement of varying IPI could introduce additional effects of stimulation on neuronal population due to non-linear responses of the neuronal membrane to stimulation. To investigate the relationship between dynamic responses of neurons and instantaneous IPI lengths, we performed both rat experiments *in vivo* and computational modeling on stimulations of efferent axon fibers of hippocampal pyramidal cells with HFS of gradually varying and randomly varying IPI. Antidromically evoked unit spikes and PSs were used to evaluate the neuronal responses directly to pulse stimulations without involving synaptic transmission.

Hippocampal structure contains high density of neurons with clear lamellar organization of neuronal elements, allowing manipulation of distinct stimulation and recording from local cells and their axons as well as evaluation of responses of neuronal populations. Additionally, the hippocampus plays an important role in brain diseases such as epilepsy and Alzheimer’s disease (Fisher and Velasco, 2014; Deeb et al., 2019). Therefore, the responses of hippocampal neurons to electric stimulation have gained increasing attention in DBS research and in clinic. In addition, we investigated the neuronal responses initiated from axons because axons are more prone to excitation by extracellular pulses than other structural elements of neurons (Nowak and Bullier, 1998). The results of this study provide new clues to design effective novel stimulation paradigms with temporal arrangement of IPI for extending the application of DBS.

## Materials and Methods

### Animal Experiments

The animal experiment was approved by the Institutional Animal Care and Ethics Committee, Zhejiang University. Seven adult Sprague-Dawley rats (250–350 g) were anesthetized with urethane (1.25 g/kg, i.p.) and placed in a stereotaxic apparatus (Stoelting Co., United States). Details of surgical procedures and electrode placements have been reported previously (Feng et al., 2013, 2014). Briefly, a 16-channel array of recording electrodes (#Poly2, NeuroNexus Technologies, United States) was inserted into the hippocampal CA1 region [anterior–posterior, −3.5; medio-lateral, 2.7; dorso-ventral, 2.5]. A stimulation electrode (#CBCSG75, a concentric bipolar electrode, FHC Inc., United States) was inserted in the CA1 alveus [anterior–posterior, −4.8; medio-lateral, 2.7; dorso-ventral, 2.3] for antidromically activating the neurons upstream in the recording site. Based on the clear lamellar organizations of neuronal structures in the hippocampus, the recordings of both unit activity and the specific waveforms of evoked potentials along the 16 channels were used to correct the final positions of the two electrodes. The channel closest to the pyramidal layer that was able to record an evoked-PS with a maximum amplitude was used to analyze the changes of evoked potential during HFS.

Electrical signals collected by the recording electrode were amplified by a 16-channel extracellular amplifier (Model 3600, A-M Systems Inc., United States) with a frequency range of 0.3–5000 Hz. The amplified signals were then sampled by an ML880 Powerlab 16/30 data acquisition system (ADInstruments Inc., Australia) at a sampling rate of 20 kHz/channel. PSs were extracted by a high-pass digital filter with a cutoff of 50 Hz.

Stimulations were sequences of biphasic current pulses with a strength of 0.3 mA and a width of 0.1 ms per phase. To compare the steady-state neuronal responses with those of two temporal arrangements of varying IPI, we utilized two patterns of pulse sequences: a same prelude 20-s period of 100-Hz HFS with constant IPI of 10 ms allowing the neuronal responses to reach steady state, followed by a 10-s period of 100–200 Hz varying IPI either quasi-linearly decreasing from 10 to 5 ms or randomly varying in the range of 5–10 ms. The mean frequency of varying IPI was 133 Hz, similar to the most used DBS frequency 130 Hz. The deceasing IPI was “quasi-linear” because it was designed to have a uniform distribution of IPI counts but not with an exact linear relationship between IPI lengths and time course.

The 10-s sequences of gradually varying IPI and randomly varying IPI were composed of the same set of 1,334 IPIs in the range of 5–10 ms with a sampling resolution of 0.05 ms, that is, 20 kHz, same as the sampling rate of the signal recordings in the experiments. Under this resolution, the 5- to 10-ms range was divided into 101 different lengths of IPI. With a uniform distribution, each IPI length was repeated 13 times in the sequences with an addition of one IPI in every five IPI lengths to get a total of 1,334 IPIs (101 × 13 + 21) with a duration of 10.005 s. The 1,334 IPIs were arranged from the longest to the shortest in the sequence of gradual IPI or were randomized by the MATLAB function randperm() to get the sequence of random IPI. Four different sequences of random IPI were used in the rat experiments, and one of the sequences was used in the computational simulation.

### Simulation Models

The computational model includes a population of pyramidal neurons in hippocampus with the mechanisms of potassium ion accumulation and clearance in the narrow peri-axonal space, distributed application of stimulation currents induced by a bipolar electrode, and recording of extracellular potential. The simulation was performed by using the NEURON simulation package (Hines and Carnevale, 1997).

#### Model of Pyramidal Neurons

##### Morphology

**FIGURE 1:**
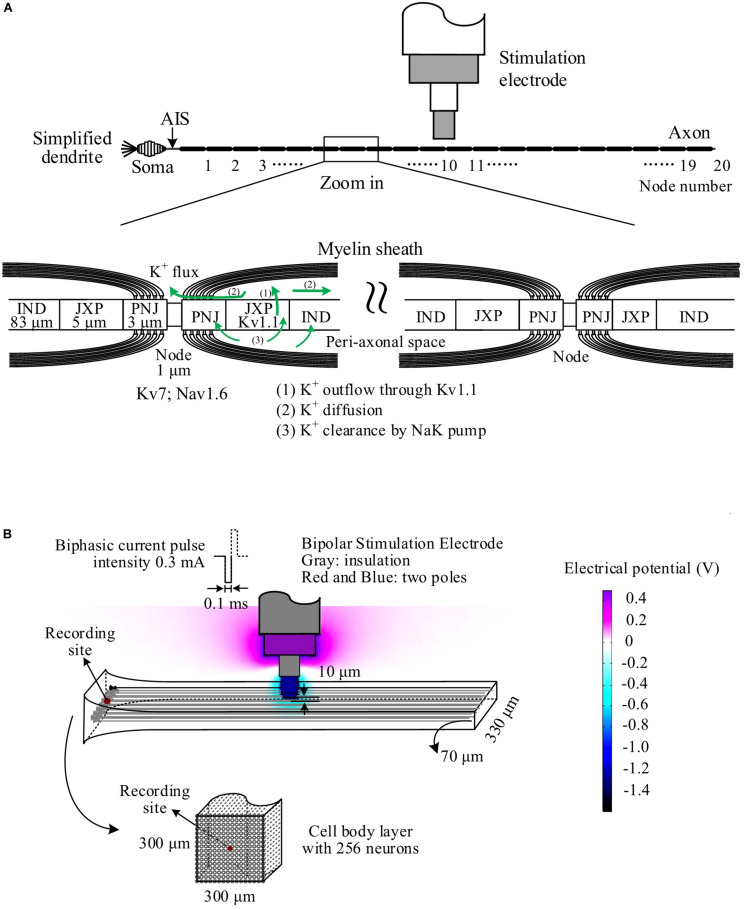
Schematic diagram of the simulation model. **(A)** The neuron consisting of a cell body, simplified dendrites, an axonal initiation segment (AIS) and a long myelinated axon. The axon consisted of 20 Ranvier nodes (Node) and 20 internode sections including paranodal junction (PNJ), juxtaparanode (JXP), and internode (IND). Voltage-gated channels of K^+^ (Kv7) and Na^+^ (Nav1.6) are on the membrane of Node, whereas fast K^+^ channels (Kv1.1) are on the JXP membrane. The green lines denote the outwards flow of K^+^ through Kv1.1 channels, accumulating in the peri-axonal space, back through NaK pump, and diffusing to the outside of Node. **(B)** Simulation of the electric field generated by the negative phase of a stimulation pulse in COMSOL. A layer of 256 neurons are arranged in parallel with small axial shifts randomly distributed within 20 μm. The stimulation electrode is located 10 μm over the surface of neuronal layer above the central node (Node_10_) of all neurons, delivering biphasic current pulses (0.1 ms, 0.3 mA). A mimic recording electrode is set at the center of cell body end.

The pyramidal neuron consisted of a cell body, an axonal initial segment (AIS), dendrites, and a long myelinated axon (Figure 1A). The cell body was an asymmetric spindle with a length of 20 μm (Migliore et al., 1999), consisting of 10 cylinder segments with a maximum diameter of 20 μm in the center that linearly decreased to 5 μm to the dendrite side and to 1.7 μm to the AIS side. The AIS was 50 μm long with a diameter of 1.22 μm (Hu et al., 2009). The dendrites were simplified as five separate cylinder segments, each with 20-μm length and 5-μm diameter. The myelinated axon consisted of 20 Ranvier nodes (abbreviated as Node) and 20 internode segments with detailed structures similar to those of a previous axon model (Bellinger et al., 2008; Guo et al., 2018). The outside diameter of axon was ∼1 μm wrapped by 15 layers of myelin membranes. The internode segments were made up of paranodal junction (PNJ), juxtaparanode (JXP), and internode (IND). Details of sizes and morphologic parameters of each neuronal element are shown in Table 1.

**TABLE 1:** Morphologic parameters of the neuron model.

**Parameter**	**Value**
**Cell body**	
Soma length	20 μm
Soma maximum diameter	20 μm
Axonal initial segment (AIS) length	50 μm
Axonal initial segment diameter	1.22 μm
**Dendrites**	
Dendritic (five segments) length	100 μm
Dendritic diameter	5 μm
**Myelinated axon**	
Axon diameter	1 μm
Number of myelin lamella	15
Node length	1 μm
Node diameter	0.7 μm
Internode segment length	100 μm
PNJ length and diameter	3 and 1 μm
JXP length and diameter	5 and 1 μm
IND length and diameter	83 and 1 μm
Peri-axonal space width outside PNJ	0.004 μm
Peri-axonal space width outside JXP and IND	0.012 μm

*PNJ, paranodal junction; JXP, juxtaparanode; IND, internode.*

##### Electrical properties

The neuronal membrane consisted of both passive and active electrical properties. The parameters of passive electrical properties include membrane capacitance, leakage conductance, leakage reverse potential, and intracellular resistivity. The capacitance and leakage conductance were distributed on the entire membrane of the neuron including the myelin. The intracellular resistivity was 300 Ω cm in cell body and 70 Ω cm in axon (McIntyre et al., 2004). The details of the passive electrical parameters are shown in Table 2.

**TABLE 2:** Electrical parameters of the cell body, dendrite, and axon.

**Parameter**	**Value**
**General**	
Membrane capacitance	1 μF/cm^2^
Leakage reversal potential	−65 mV
Na^+^ Nernst potential	45 mV
K^+^ Nernst potential (without K^+^ accumulation)	−90 mV
Ca^2+^ Nernst potential	140 mV
**Soma and dendrite**	
Max voltage-gated Na^+^ conductance	80 pS/μm^2^
Max slow non-inactivating K^+^ conductance	0.3 pS/μm^2^
Max fast voltage-gated K^+^ conductance	20 pS/μm^2^
Max Ca^2+^-dependent K^+^ conductance	3 pS/μm^2^
Max high-voltage activated Ca^2+^ conductance	0.3 pS/μm^2^
Leakage conductance	0.5 pS/μm^2^
**Axonal initial segment (AIS)**	
Max fast voltage-gated Na^+^ conductance	1,100–3,200 pS/μm^2^
Max fast voltage-gated K^+^ conductance	200–1,000 pS/μm^2^
Leakage conductance	0.5 pS/μm^2^
**Axon internode section**	
Myelin conductance	10 pS/μm^2^
Myelin capacitance	0.1 μF/cm^2^
PNJ leakage conductance	1 pS/μm^2^
JXP leakage conductance	1 pS/μm^2^
JXP max fast K^+^ conductance	300 pS/μm^2^
IND leakage conductance	1 pS/μm^2^
**Axon node**	
Max voltage-gated Na^+^ conductance	6,000 pS/μm^2^
Max slow voltage-gated K^+^ conductance	150 pS/μm^2^
Leakage conductance	1 pS/μm^2^

*PNJ, paranodal junction; JXP, juxtaparanode; IND, internode.*

The parameters of active electrical properties including sodium, potassium, and calcium channels are distributed in different membrane sections (Table 2 and Figure 1A). The ion channels distributed on the neuronal membrane include the followings: voltage-gated Na^+^, non-inactivating K^+^ (M current) channel, voltage-gated K^+^ channel, Ca^2+^-dependent K^+^ channel, and high-voltage activated Ca^2+^ channel for cell body and dendrites; voltage-gated K^+^ channel (Kv) and voltage-gated Na^+^ channel (Nav1.2 and Nav1.6) for the AIS (Hu et al., 2009); fast K^+^ channel (i.e., Kv1.1) for the membrane of JXP in the internode sections; and voltage-gated Na^+^ channel (Nav1.6) and slow K^+^ channel (Kv7) for the Node. Specifically, the Nav1.2 subunit was present in proximal AIS, whereas Nav1.6, although present in proximal AIS, was dominant in distal AIS and the axonal Node. The Kv density increased linearly with distance in the AIS to a maximum value of 1,000 pS/μm^2^. A previous model (Hu et al., 2009) may be referred to for the detailed distribution of Na^+^ and K^+^ channels in the AIS. On the Node membrane, the density of Nav1.6 channels was about 6,000 pS/μm^2^ (Lorincz and Nusser, 2010) and the K^+^ channel density was 150 pS/μm^2^ (Röper and Schwarz, 1989; Battefeld et al., 2014).

#### Accumulating and Clearing Mechanisms of Potassium Ions in the Model

Potassium accumulated in the narrow peri-axonal space during stimulation because of the outflow of K^+^ from the intracellular space through K^+^ channels in JXP (Bellinger et al., 2008; Guo et al., 2018). The accumulated K^+^ was cleared by NaK pumps distributed uniformly on the membrane of internode sections and by axial diffusion of K^+^ from the peri-axonal space to the extracellular space outside Nodes (indicated by the green arrow in Figure 1A). The axial diffusion of K^+^ followed the Fick law:

(1) $$J=D\times A\times \frac{\text{d}\,{\left[{\text{K}}^{+}\right]}_{\text{o}}}{\text{d}\,x}$$

where *J* is the diffusion flux; *D* is the diffusion coefficient (1.85 μm^2^/ms); *A* is the cross-sectional area of the peri-axonal space of 0.025, 0.076, and 0.076 μm^2^ outside PNJ, JXP, and IND, respectively; [K^+^]_*o*_ is the K^+^ concentration in peri-axonal space; and *x* is the diffusion distance. The concentration of potassium ion outside the Node (extracellular concentration) was 3 mM.

The clearance of K^+^ by NaK pump, which pumped out three Na^+^ and pumped in two K^+^ per ATP hydrolyzed, was described as follows:

$$I_{\text{NaK}}=I_{\text{NaK}\,\text{max}}\,\left(\frac{{\left[{\text{K}}^{+}\right]}_{\text{o}}}{{\left[{\text{K}}^{+}\right]}_{\text{o}}+\text{KmK}}\right)$$

(2) $$\;\times \left(\frac{{\left[{\text{Na}}^{+}\right]}_{\text{i}}^{1.5}}{{\left[{\text{Na}}^{+}\right]}_{\text{i}}^{1.5}+{\text{KmNa}}^{1.5}}\right)\,\left(\frac{V+150}{V+200}\right)$$

where *V* is the membrane potential; [Na^+^]_*i*_ is intracellular Na^+^ concentration; *I*_*NaKmax*_ is the maximum transport current per unit area, set as 2.46 μA/cm^2^; and KmK and KmNa are the equilibrium binding constants of K^+^ and Na^+^, set as 5.3 and 27.9 mM, respectively.

#### Models of Stimulation and Recording

Pulse sequences were applied by a stimulation electrode located 10 μm above the top surface of the axial center of an axon layer or approximately over the center Nodes (Node_10_) of axons (Figure 1A). The layer included 256 axons distributed uniformly in an array of 8 × 32 in a cross section of 70 μm × 310 μm with an inter-axon distance of 10 μm in both spatial orientations (Figure 1B). The axial positions of the parallel axons shifted randomly within a range of −10 to +10 μm, mimicking the lamellar organization of alveus axons. The cell bodies of the neurons were distributed uniformly in an array of 16 × 16 in a cross section of 300 μm × 300 μm and in random one-to-one correspondence with the 256 axons.

The electrical field generated by biphasic pulses was simulated with finite element software COMSOL Multiphysics 5.3 (COMSOL Inc., Sweden), thereby determining the stimulation potentials imposing immediately outside every Ranvier node (Node) of the axonal fibers. Both the electrode parameters (size and material) and stimulation parameters (current biphasic pulses) used in COMSOL simulation were consistent with experimental settings. The stimulation sequences applied in the model were similar to those used in rat experiments, that is, following a prelude of 100-Hz HFS with constant IPI by 100–200 Hz varying IPI of either gradual IPI or random IPI. A recording site was located in the center of the cell body layer to record extracellular potential (Ep) including the extracellular action potentials either from a specific single neuron (i.e., unit spike, abbreviated as spike) or from a population of neurons (i.e., PS). The equation for Ep was as follows (Varona et al., 2000; Guo et al., 2018):

(3) $$\text{Ep}=\frac{1}{4\,\pi \,\sigma }\,\sum \frac{I}{r}$$

where σ is the extracellular conductivity (0.286 S/m), *I* is the transmembrane current, and *r* is the distance between the recording site and each neuronal element generating *I*, including the compartments of cell body and AISs. Although the transmembrane currents from dendrites may contribute to the shape of the PS waveforms (Varona et al., 2000), we did not take into account the dendrite currents because the exact PS waveform was not critical for the study. We only utilized the PS amplitude, especially the relative changes of PS amplitudes during an HFS sequence, to estimate the relative changes in the number of firing neurons. The major contributions from cell body and AIS were adequate.

Other signals such as intracellular membrane potentials (*V*_*m*_), concentration of K^+^ in peri-axonal spaces ([K^+^]_*o*_), and dynamics of Na^+^ channels (e.g., inactivation variable of Na^+^ channel, h_Na) were extracted directly from simulation results in NEURON.

### Data Analyses for Both Rat Experiments and Modeling

The amplitude of PS was used to evaluate the number of neurons simultaneously generating action potentials (Kloosterman et al., 2001). The PS amplitude was normalized by the mean PS amplitude induced during steady-state of HFS with constant IPI of 10 ms. The mean value of normalized PS amplitudes induced by each pulse during varying IPI was calculated by dividing the sum of PS amplitudes by the pulse number. The range (maximum–minimum) of normalized PS amplitudes was used to describe the variation of PS amplitudes. Because the history of IPIs plays a role in how many neurons are recruited in the PS, the two IPIs immediately preceding a stimulus (“1-back IPI” and “2-back IPI,” termed as IPI_1_ and IPI_2_, respectively) were examined. Correlation analysis was implemented to analyze the relationships between the PS amplitude and the preceding IPIs (including IPI_1_ and ΔIPI = IPI_1_−IPI_2_). Student *t*-test was used to show the statistical significance of the difference between data groups. Additionally, in simulation, the threshold of h_Na at the center node (Node_10_) for a specific neuron was defined as the minimal value of h_Na at the onset of a pulse that was able to induce an action potential successfully propagating to the cell body.

## Results

### Different Neuronal Responses to High-Frequency Stimulation With Gradually and Randomly Varying Inter-Pulse Interval in Rat Hippocampus *in vivo*

**FIGURE 2:**
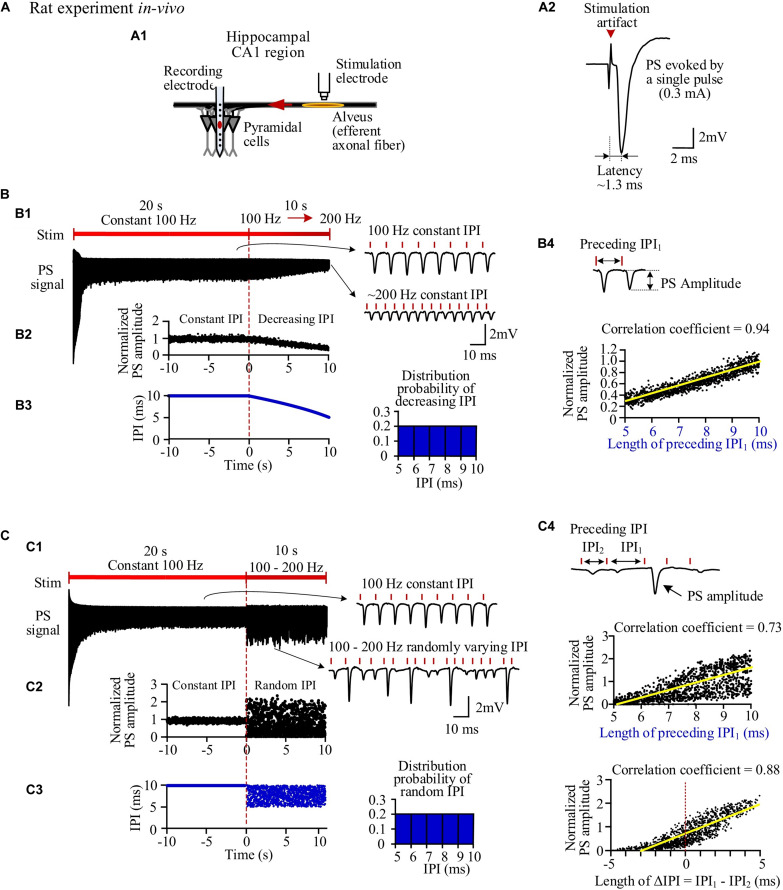
Neuronal responses to stimulations with different inter-pulse interval (IPI) sequences in rat hippocampal CA1 region *in vivo*. **(A)** A1: Schematic diagram of the locations of a recording electrode in pyramidal layer and a stimulation electrode at alveus to antidromically activate the pyramidal cells. A2: A population spike (PS) evoked by a single pulse (0.3 mA). **(B)** PS evoked by pulses with gradually decreasing IPI. B1: A typical PS recording during 30-s HFS consisted of 20 s of constant IPI (10 ms, 100 Hz) followed by 10 s of gradually decreasing IPI (10 to 5 ms, i.e., 100 to 200 Hz). B2: Changes of normalized PS amplitudes during 10 s of constant IPI and 10 s of gradual IPI. The switch moment from constant IPI to gradual IPI is set as time “0.” B3: IPI curve along stimulation time (*left*) and distribution probability of gradual IPI (*right*). B4: Correlation between normalized PS amplitude and preceding IPI during 10-s period with gradual IPI. **(C)** PS evoked by pulses with random IPI. C1–C4: Corresponding to B1–B4. *Bottom of* C4: Correlation between the normalized PS amplitude and the difference of IPI_1_–IPI_2_.

Stimulations applied in the alveus of hippocampal CA1 region can antidromically excite a population of CA1 pyramidal neurons without involving synaptic transmissions (Figure 2A1). A single pulse evoked a large PS (Figure 2A2) with a short latency of 1.27 ± 0.09 ms and an amplitude 9.1 ± 1.9 mV (*n* = 7 rats), just as that evoked by the very first pulse in any pulse sequences. However, after seconds of prolonged 100-Hz HFS with constant IPI, during the steady state, each pulse only evoked a small PS with an amplitude ∼20% of the original amplitude. Afterward, when the IPI gradually decreased from 10 ms (100 Hz) to 5 ms (200 Hz) in a 10-s period, the PS amplitude decreased further to ∼6% of the original amplitude (Figure 2B1). The PS amplitudes induced by gradual IPI were compared with those with constant IPI in a 10-s period immediately before the gradual IPI (Figure 2B2, the switch moment of the two IPI patterns is set as time “0,” with the time duration of −10 to 0 s for constant IPI and 0–10 s for gradual IPI). The normalized PS amplitude decreased from 1 to 0.3 during the 10-s period of gradual IPI. During this period, the IPI decreased quasi-linearly and distributed uniformly in the range of 5–10 ms (Figure 2B3). The PS amplitude significantly correlated with its 1-back IPI (IPI_1_) with a correlation coefficient of 0.94 (Figure 2B4). When the above stimulation was followed by a pulse sequence with a reverse change of IPI from 5 to 10 ms, a reverse change of PS increase was observed, as expected. For simplicity, the data are not repeatedly presented here.

When the varying IPI of a gradual order was replaced by a random order, the normalized amplitudes of evoked PS varied randomly in a much wider range from 0 (no PS) to ∼2.4 (Figures 2C1,C2), as compared with the range of 0.3–1 induced by gradual IPI. Although the random IPI was in the same range of 5–10 ms and with the same uniform distribution (Figure 2C3), the correlation coefficient (0.73) between the PS amplitudes and the preceding IPI_1_ of random IPI was smaller than that of gradual IPI. The less correlation was caused by a lot of small PSs appearing following the longer IPIs (Figure 2C4). However, the correlation coefficient between the PS amplitudes and ΔIPI (=IPI_1_−IPI_2_) increased to 0.88 (Figure 2C4), indicating that a longer IPI_1_ accompanied by a short IPI_2_ (2-back IPI) would result in a larger PS with a higher probability.

Statistical data (*n* = 7 rats) showed that the mean normalized PS amplitude during 10-s period of random IPI was similar to that of gradual IPI (Table 3), whereas the range of normalized PS amplitude with random IPI was significantly greater than the value with gradual IPI. However, because of the same pulse number and similar mean of normalized PS amplitude, the sums of normalized PS amplitude during gradual IPI and random IPI were approximately equal, indicating similar amounts of total neuronal firing. Additionally, the mean correlation coefficient between the PS amplitude and the preceding IPI_1_ (or IPI_1_−IPI_2_) was significantly smaller with random IPI than with gradual IPI. The results of large PS indicated that pulses with random order of varying IPI could occasionally recruit a larger fraction of neurons to fire together.

**TABLE 3:** Normalized PS amplitudes distribution during gradually and randomly varying IPI.

**IPI patterns**	**Normalized PS amplitude**		**Correlation coefficient (PS amplitude vs. IPI)**	
	**Range**	**Mean**	**Preceding IPI_1_**	**Preceding IPI_1_−IPI_2_**
**Experiment (*n* = 7 rats)**				
Gradual	0.92 ± 0.05	0.72 ± 0.06	0.92 ± 0.03	–
Random	2.49 ± 0.43**	0.77 ± 0.06	0.72 ± 0.04**	0.84 ± 0.03^##^
**Simulation**				
Gradual	1.12	0.62	0.92	–
Random	2.95	0.73	0.73	0.8

PS, population spike; IPI, inter-pulse interval. **P < 0.001, gradual IPI vs. random IPI, *t*-test, *n* = 7 rats. ^##^*P* < 0.001, random IPI_1_-IPI_2_ vs. random IPI_1_, *t*-test, *n* = 7 rats.

Previous studies with constant IPI have shown that prolonged HFS can extend the refractory periods of the neuronal membranes owing to accumulation of extracellular potassium, leading to the intermittent firing of individual neurons and a decrease of PS amplitude with higher frequency (i.e., shorter IPI) (Feng et al., 2014; Guo et al., 2018). We hypothesize that the same mechanism of potassium accumulation in the narrow peri-axonal space together with non-linear dynamics of ionic channels may generate the irregular neuronal responses with simultaneous recruitment of a larger fraction of neurons by pulses with random order of varying IPI. Because current techniques of *in vivo* experiments have limitations in simultaneous intracellular recordings of individual axons, we next tested the hypothesis by a modeling study.

### Simulated Neuronal Responses to High-Frequency Stimulation With Varying Inter-Pulse Interval of Gradual and Random Orders

**FIGURE 3:**
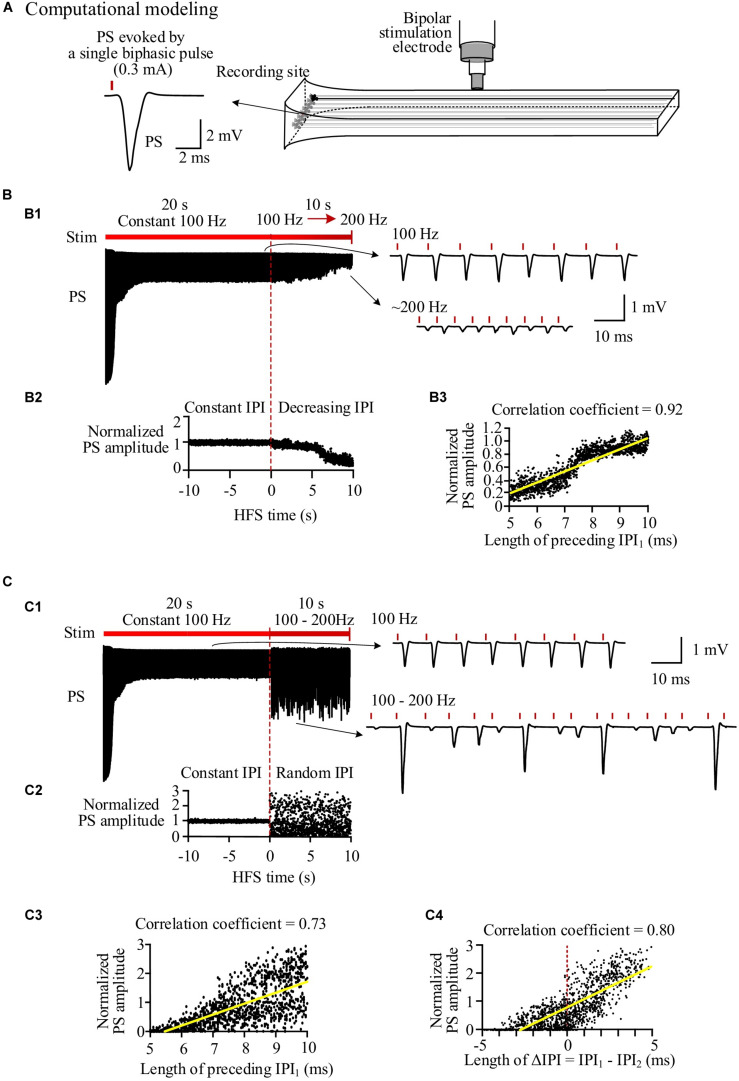
Simulation of neuronal responses to stimulations with different inter-pulse interval (IPI) sequences. **(A)** Schematic diagram of the locations of stimulation electrode and recording electrode together with a population spike (PS) waveform evoked by a single pulse. **(B)** Simulated PS evoked by pulses with gradual IPI. B1: Simulated PS signal during 30-s HFS including 20 s of constant IPI (10 ms, 100 Hz) followed by 10 s of gradually decreasing IPI (10 to 5 ms, i.e., 100 to 200 Hz). B2: Changes of normalized PS amplitudes during 10 s of constant IPI and 10 s of gradual IPI. The switch moment from constant IPI to gradual IPI is set as time “0.” B3: Correlation between normalized PS amplitude and its preceding IPI during 10-s period with gradual IPI. **(C)** Simulated PS evoked by pulses with random IPI. C1–C3: Corresponding to B1–B3. C4: Correlation between the normalized PS amplitude and the difference of IPI_1_–IPI_2_.

The simulated evoked PS (Figure 3) closely matched the experimental recordings (Figure 2). At the onset of a pulse sequence, the evoked PS was also as large as a PS evoked by a single pulse (Figures 3A,B1). Following seconds of prolonged 100-Hz HFS, during the steady-state period, the PS amplitude decreased to ∼20% of the original amplitude. Afterward, when IPI gradually decreased from 10 to 5 ms in a 10-s period, the PS amplitude further decreased to ∼4% of the original amplitude (Figure 3B1). The normalized PS amplitude decreased from ∼1 at the start (corresponding to 10-ms IPI) to ∼0.2 at the end (corresponding to 5-ms IPI) during the 10-s period with gradual IPI (Figure 3B2). In addition, the PS amplitude was significantly correlated with its preceding IPI with a correlation coefficient of 0.92 (Figure 3B3 and Table 3).

Similar to the experimental results, when the varying IPI in a gradually decreasing order was replaced with a random order, the normalized PS amplitude varied in a much wider range from 0 to 2.95 (Figures 3C1,C2), more than double of the value with gradual IPI (2.95 vs. 1.12, Table 3). However, the mean normalized PS amplitudes during the periods with two different orders of varying IPI were relatively close (0.62 vs. 0.73). Additionally, the correlation coefficient between the PS amplitude and the preceding IPI_1_ was smaller with random order IPI than with gradual IPI (0.73 vs. 0.92); but the correlation coefficient between the PS amplitude and the ΔIPI increased to 0.8 (Figures 3C3,C4 and Table 3).

The PS only reflects the summation of firing from a population of neurons. To investigate the underlying mechanisms, we next examined unit spike firing of individual neurons.

### Simulated Membrane Dynamics of Individual Neurons During High Frequency Stimulation With Different Inter-Pulse Interval Patterns

**FIGURE 4:**
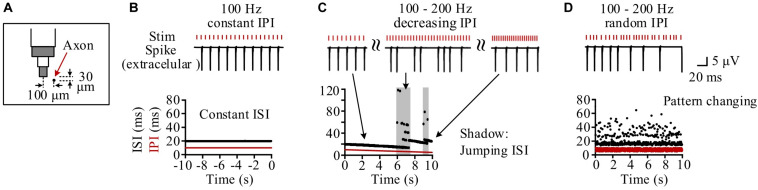
Simulation of the response firing of a typical neuron to different pulse sequences. **(A)** Schematic diagram of the neuron’s location with its axon center (Node_10_) 104.4 μm (30 μm below and 100 μm left) from the bottom center of the stimulation electrode. **(B)** During the steady-state period of constant inter-pulse interval (IPI) of 10 ms, the neuron fires with a constant inter-spike interval (ISI) of 20 ms. **(C)** During the stimulation period with gradual IPI, the ISI of neuronal firing gradually decreases along with the decrease of IPI in most time except several jumping stages (denoted by shadows) to elevate the ISI in stairs by adding one or more IPI into ISI. **(D)** During the stimulation period with random IPI, the ISI of neuronal firing changes irregularly all the time.

Neurons at different distances from the stimulation electrode had different periodical firing patterns because they received different stimulation intensities (Guo et al., 2018). We took one neuron as an example, with its middle axonal node (Node_10_) located at a distance 104.4 μm (30 μm below and 100 μm left) from the stimulation center (Figure 4A). During the steady state of 100 Hz of constant HFS, the inter-spike interval (ISI) of the neuronal firing kept constant at two times the IPI as the neuron fired a spike at every other pulses (Figure 4B). During the period of gradually decreasing IPI, the firing ISI exhibited two alternating stages: gradually decreasing stage and jumping stage (Figure 4C). At a gradually decreasing stage, the ISI decreased gradually following the decrease of IPI as the neuron fired steadily at every *n*-th pulses. The stage was interrupted by sporadic and short jumping stages when the ISI increased by one or more IPI. Therefore, during most of the stimulation period with gradual IPI, the firing of individual neurons was similar to the firing under constant IPI. However, during the entire 10-s stimulation period with random IPI, the firing ISI changed randomly in a wider range (14.5–64.5 ms) (Figure 4D).

**FIGURE 5:**
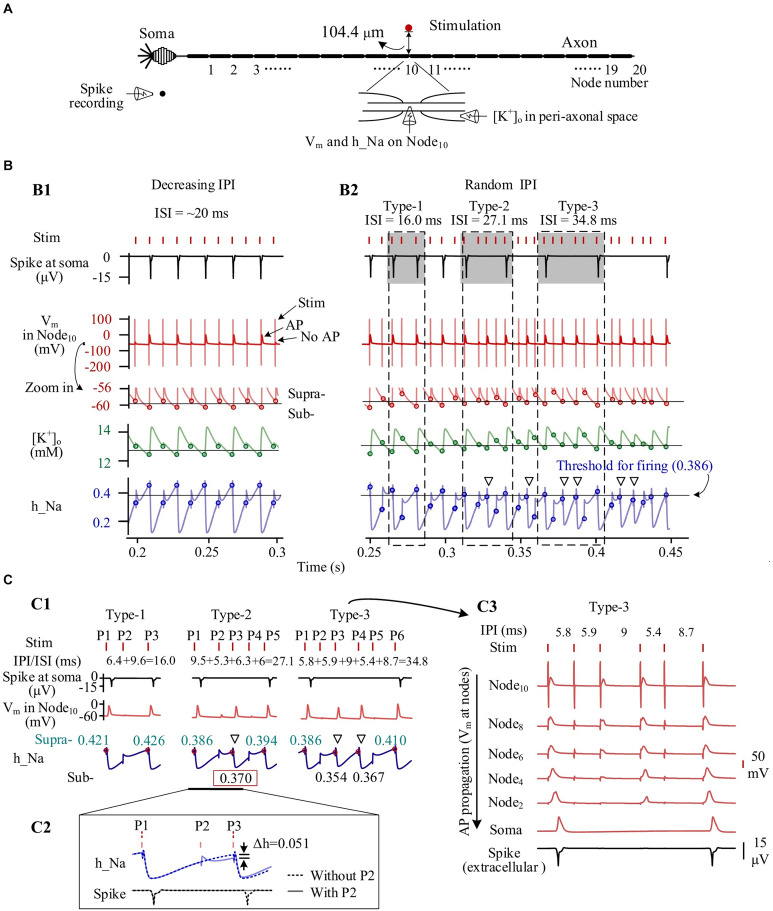
Simulated dynamics of neuronal membrane during high-frequency stimulation (HFS) with different patterns of inter-pulse interval (IPI). **(A)** Schematic diagram of simulated signals collected from a neuron, including K^+^ concentration in peri-axonal space near Node_10_ ([K^+^]_o_), membrane potentials of Node_10_ (*V*_m_), inactivation variable of Na^+^ channels on Node_10_ (h_Na), and spike recording near soma. **(B)** Dynamics of four signals during stimulations with gradually varying IPI (B1) and randomly varying IPI (B2). Circles on the curves of *V*_m_, [K^+^]_o_ and h_Na denote the values at the onset of stimulation pulses. Thin horizontal lines on the three curves denote thresholds for neuronal firing. Three typical IPI sequences (shadowed) generate different lengths of inter-spike interval (ISI) of 16.0, 27.1, and 34.8 ms. **(C)** Generation of different ISI. C1: The three examples of IPI sequences from B2 show that the levels of inactivation variable h_Na at the onset of pulses determine whether AP can be induced at Node_10_ and whether the AP can successfully propagate to soma. The red dots and cyan numbers on the h_Na curves denote h_Na values at the onset of pulses inducing AP at Node_10_. C2: A pulse (P2) failing to induce AP can decrease h_Na and delay the recovery course of h_Na, thereby causing no spike at soma following the next pulse (P3). A spike would otherwise be evoked by P3 in the situation without P2. C3: Even an AP is induced on Node_10_ immediately under the stimulation electrode, due to an inadequate recovery of h_Na, the AP may not be strong enough to propagate along the axon to generate a spike at soma. Hollow triangles in B2 and C1 denote that the APs of Node_10_ fail to generate a somata spike but can cause a substantial decrease of h_Na.

To analyze the mechanisms underlying the behaviors of individual neurons during HFS with gradual IPI and random IPI, we extracted the following variables from the simulation results: the accumulated [K^+^]_*o*_ in peri-axonal space near Node_10_, the membrane potential (*V*_*m*_) on Node_10_, the inactivation variable (h_Na) of Na^+^ channel on Node_10_, and the extracellular spike signal of the soma (Figure 5A).

Following the prelude 20-s period of 100-Hz HFS with constant IPI of 10 ms, the [K^+^]_*o*_ had increased from original 3 to above 12 mM. The accumulated K^+^ elevated *V*_*m*_ from original −70 to around −60 mV, thereby inactivating Na^+^ channel as h_Na declining from baseline value 0.95 to below 0.5. Nodal membrane depolarized upon each incoming pulse and repolarized during the interval before the incoming of next pulse.

During most of the period with gradual IPI, pulses of HFS changed the membrane dynamics of the neuron periodically. Figure 5B1 shows an episode of gradually decreasing IPI of ∼10 ms. At every other pulse, an action potential (AP) was generated and then resulted in a pronounced elevation of [K^+^]_*o*_ as well as a pronounced decline of h_Na, consequently. Accordingly, the *V*_*m*_, [K^+^]_*o*_ and h_Na changed periodically. At the onset moment of each pulse, *V*_*m*_ on Node_10_ was either −59.0 or −60.6 mV, [K^+^]_*o*_ 12.9 or 12.4 mM, and h_Na 0.330 or 0.454, respectively corresponding to a subthreshold state or a suprathreshold state of firing. The alternation between the two states gave rise to an intermittent and regular firing of spikes with an ISI of ∼20 ms, two times the IPI.

In contrast, HFS with random IPI (5–10 ms) induced irregular firing of spikes in the same neuron owing to the non-linear dynamics of the membrane excitation. Because the inactivation of Na^+^ channel (h_Na) has a much longer time constant than the activation of Na^+^ channel (Hodgkin and Huxley, 1952), the excitability of Na^+^ channels during HFS was mainly determined by the recovery of h_Na. A small difference in h_Na could change the membrane excitability and flip its state from subthreshold to suprathreshold or vice versa, thereby drastically changing the firing pattern of the neuron (Figure 5B2). For instance, for the three firing patterns of type 1, type 2, and type 3 illustrated in Figures 5B2,C1, one, three, and four pulses failed to activate spikes in soma and resulted in an ISI of 16.0, 27.1, and 34.8 ms, respectively. Failed pulses still caused the membrane depolarization that delayed the recovery course of h_Na. In this case, the h_Na threshold for generating a spike at soma was 0.386 (Figure 5B2). For the type 2 firing pattern shown in Figure 5C1, with a preceding pulse P2 (a failed pulse), a recovery of h_Na to 0.370 at the onset moment of pulse P3 only allowed the pulse to generate an AP at Node_10_, but the AP failed to activate the soma to fire a spike. Otherwise, in a case without the preceding failed pulse P2, the h_Na would have recovered to 0.421 to exceed the threshold, thereby driving the soma to fire a spike (Figure 5C2).

It should be noted that a fraction of AP induced at axonal nodes were not strong enough to travel to the soma and generate an AP (i.e., a spike) at soma (see Figures 5B2,C1). Nevertheless, the failed AP would decrease h_Na substantially, delaying the recovery of h_Na and possibly preventing the next pulse from generating a spike (denoted by hollow triangles in Figures 5B2,C1). The repeated delays of h_Na recovery resulted in a prolonged interval between two adjacent spikes (Figure 5C3).

These simulation results indicated that stimulations with regular IPI (including most of the period with gradually varying IPI) can generate AP with regular ISI in individual neurons. However, stimulations with a random order of varying IPI can postpone the firing of individual neurons and generate AP with various ISI. The different patterns of ISI could result in different numbers of neurons to fire spikes simultaneously. Therefore, we next investigated the firing of neuron populations by modeling.

### Comparing Firing of Neuronal Population Induced by High-Frequency Stimulation With Regular and Random Inter-Pulse Interval

**FIGURE 6:**
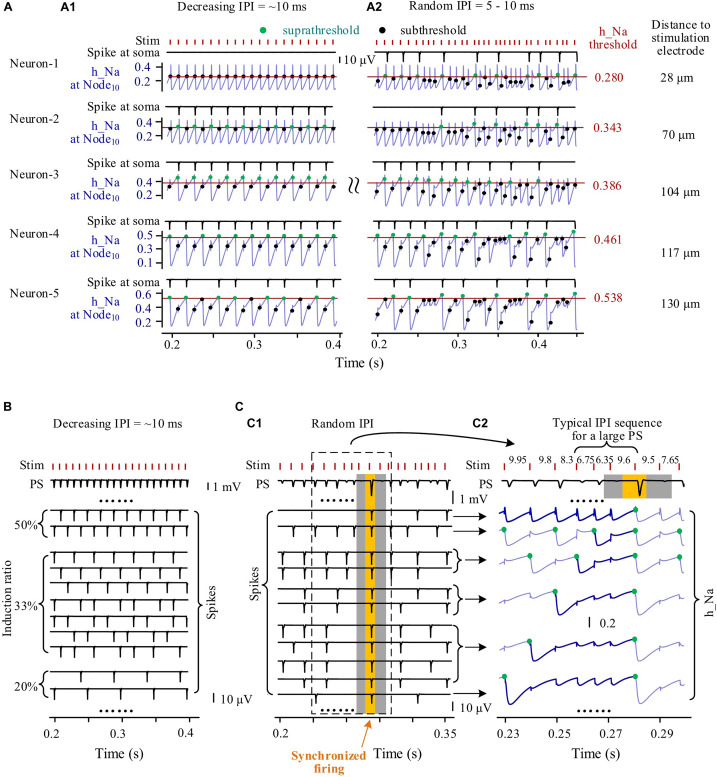
Simulated responses of multiple neurons to form population spikes (PSs) during stimulations with different patterns of inter-pulse interval (IPI). **(A)** Somata spikes and inactivation variable h_Na curves of five neurons with different distances (28–130 μm) from the stimulation electrode. Thin horizontal lines on the h_Na curves denote spike thresholds of individual neurons (0.280–0.538). A1: During an episode of stimulation with gradually decreasing IPI, except that Neuron-1 does not fire spikes, the other four neurons generate spikes regularly with h_Na regularly switching between suprathreshold and subthreshold levels (denoted by green and black dots, respectively) upon a pulse incoming. A2: During stimulation with random IPI, all of the five neurons generate spikes irregularly with h_Na surpassing threshold occasionally upon a pulse incoming. **(B)** During an episode of stimulation with gradually decreasing IPI, regular firing of individual neurons with different induction ratios (ratio of spike number to pulse number) produces uniform PS. **(C)** During stimulation with random IPI, a large PS formed by synchronous neuronal firing is induced by a typical IPI sequence. C1: Different spike sequences of individual neurons integrate into PS sequences with various PS amplitudes including a large PS (yellow zone) with surrounding silent periods of no PS (gray zones). C2: Inactivation variable h_Na curves show the recovery course of h_Na before synchronous firing of neurons following an IPI sequence including two shorter IPI (6.75 and 6.35 ms) and one longer IPI (9.6 ms). The thickening and dark-blue segments of h_Na curves show that the series of preceding pulses fail to generate somata spikes in individual neurons before the generation of the large PS.

During episodes of stimulation with gradually varying IPI, individual neurons fired spikes in a fixed periodical pattern. For example, five neurons with different distances (at Node_10_) to the stimulation center of 28, 70, 104, 117, and 130 μm had different periodical patterns of spike firing (Figure 6A1). Neurons located nearer to the stimulation electrode received a greater stimulation intensity and had a lower h_Na threshold for firing. Because of the overstimulation received, the nearest neuron (Neuron-1) was not able to fire spike, with its h_Na at Node_10_ only fluctuating below the firing threshold at the onset of each incoming pulse. The three neurons at other distances, Neuron-2, Neuron-3, and Neuron-4 fired spikes at a same induction ratio of 50% (i.e., every other pulse induced a spike), but Neuron-2 and Neuron-3 followed the same pulses whereas Neuron-4 followed the others. The farthest neuron (Neuron-5) had an induction ratio of 33%.

However, during the stimulation with random IPI, the five neurons all fired spikes irregularly with their h_Na at Node_10_ exceeding respective threshold occasionally (Figure 6A2). Interestingly, Neuron-1 changed from no firing with gradual IPI into intermittent firing with random IPI.

The differences in spike firing of individual neurons resulted in different responses of a neuronal population to different patterns of varying IPI. During an episode of stimulation with gradually varying IPI (Figure 6B), the periodical spikes of individual neuron firing were integrated to form PSs with similar amplitudes following every pulse. However, during stimulation with random IPI, owing to the irregular firing of individual neurons, the amplitudes of integrated PS varied randomly in a large range (Figure 6C1). Repeatedly decreasing h_Na by several successive pulses with shorter IPI could postpone the time for h_Na to recover to its threshold (Figure 6C2, thick blue lines in h_Na curves), finally giving rise to simultaneous firing of a large number of neurons to form a large PS following a longer IPI (the yellow zone in Figure 6C). The large PS was accompanied by very small PS induced by neighboring pulses (the two gray zones in Figure 6C). The mean PS amplitudes induced by gradual IPI and by random IPI were similar (Table 3 simulation), indicating that randomization of IPI could only redistribute the firing timing of neurons but not significantly change their firing amount.

These simulation results indicated that the stimulation pulses with random IPI could recruit neurons to fire at specific moments because of the occasional occurrence of specific sub-sequences of pulses, thereby generating extremely large and extremely small PS events.

## Discussion

The novel finding of the present study with experiments and computation is that the HFS pulses with varying IPI in random order had additional recruitment effects on the firing of neuronal population. Possible mechanisms underlying the experimental finding are unveiled by our simulation study and analyzed below.

### Possible Cellular Mechanisms Underlying the Firing Patterns of Neurons Induced by Different Patterns of Varying Inter-Pulse Interval

Previous experiment studies both *in vitro* and *in vivo* have shown that HFS with a constant frequency over 50 Hz can induce partial depolarization block of axons in the brain regions such as the hippocampus and subthalamus (Jensen and Durand, 2009; Zheng et al., 2011; Rosenbaum et al., 2014). Our previous study has shown that with a substantial decrease of antidromic PS induced by HFS applied to efferent fibers of CA1 neurons as shown in the present study, the neuronal somata preserved the capacity to respond to an orthodromic stimulus with large PS (Feng et al., 2013), thereby confirming the HFS-caused failures in the axons, not in the soma. *In vitro* studies have shown that repeated pulse stimulation can elevate [K^+^]_*o*_, thereby causing a decreased speed of axonal conduction until a complete block. Artificially increasing [K^+^]_*o*_ can also lead to axonal block (Förstl et al., 1982; Poolos et al., 1987). Computational models on HFS with constant IPI suggest the underlying mechanisms of the block: the outflow of K^+^ during stimulations may be trapped in the narrow peri-axonal space and increasing [K^+^]_*o*_, thereby prolonging depolarization of membrane and inactivation of Na^+^ channels and leading to intermittent depolarization block (Bellinger et al., 2008; Guo et al., 2018). HFS at the commonly used frequency range (∼100–200 Hz) for DBS can only induce partial but not complete axonal block. The axons can still intermittently generate action potentials to form PS events (Jensen and Durand, 2009; Feng et al., 2013, 2014). During an HFS sequence with a higher frequency (i.e., shorter IPI), each pulse can recruit fewer neurons to fire, thereby only generating a smaller PS (Guo et al., 2018).

The above mechanisms may explain the IPI-dependent PSs evoked by pulses with gradually varying IPI in the present study, as shown by the highly linear correlation between PS amplitudes and lengths of immediate preceding IPI (1-back IPI) in both the experiment and simulation results (Figures 2B, 3B and Table 3). Based on these mechanisms, it seems reasonable to speculate that the IPI-dependent PSs would have also appeared with IPI arrangements in any other orders as long as the IPI set was the same. However, our study shows that even with the same set of IPI varying only in a small range of 5–10 ms (i.e., 200–100 Hz), substantially different responses of population neurons appeared during HFS with random IPI (Figures 2C, 3C and Table 3). Here, we reveal the mechanisms of these different neuronal responses by the recovery course of Na^+^ channel with non-linear dynamics.

Owing to the much longer time constant of Na^+^ channel inactivation than that of Na^+^ activation (Hodgkin and Huxley, 1952), under the situation of intermittent depolarization block, the excitability of Na^+^ channels in response to HFS pulses would be mainly determined by the non-linear recovery of Na^+^ inactivation (h_Na). An IPI in the range of 5–10 ms was not long enough for a complete recovery of h_Na after a firing spike under the situation of HFS. It took several IPIs for h_Na to recover to a suprathreshold level. In addition, each failed pulse would also lower the h_Na and postpone the recovery of h_Na (Figures 5, 6). The firing history of a neuron, the lengths of several preceding IPIs, and the pulse impulses following the previous firing affect the non-linear recovery course of h_Na, hence the next firing of the neuron.

During HFS periods with regular IPI (e.g., gradually varying IPI or constant IPI), although the number of IPI cycles needed for the h_Na recovery varied for neurons at various distances from the stimulation point, the firing of individual neurons was regular at most time because of the approximately fixed IPI length within a relative short episode. The integration of regular firing from a large population of neurons remained steady over time (Figures 6A1,B). However, when the varying IPI appeared randomly, the number of IPI cycles as well as the sum of IPI lengths for h_Na to recover to suprathreshold was uncertain, and the firing of individual neurons became irregular, thereby occasionally generating extremely large and extremely small PSs as a result of the firing integration of a neuronal population (Figures 6A2,C).

Taken together, through the mechanisms of intermittent [K^+^]_o_ elevation and Na^+^ channel inactivation induced by HFS, as well as the non-linear recovery course of Na^+^ channel, HFS with different patterns of varying IPI in a small range can modulate the firing of neuronal populations differently. To our knowledge, this is the first simulation study addressing the effect of irregular pulse patterns on the neuronal firing by incorporating the mechanism of submyelin K^+^ accumulation and non-linear gating dynamics of Na^+^ channel activation.

### Implications of the Neuronal Firing Induced by Different Patterns of Varying Inter-Pulse Interval

Pulse frequency is the most commonly used parameter to adjust the effects of DBS applications. For example, different temporal patterns of stimulation with varying frequency (IPI) have been investigated to modulate central nervous system arousal in intact mice (Quinkert et al., 2010; Quinkert and Pfaff, 2012) and in mice with traumatic brain injury (Tabansky et al., 2014) and to suppress epileptogenesis (Santos-Valencia et al., 2019). Irregular temporal patterns of stimulation clearly offer the opportunity to improve the efficacy and efficiency of DBS therapies (Grill, 2018). However, the vast design space for arbitrary sequences of varying IPI poses a clear challenge in designing irregular temporal patterns of stimulation. It is not clear how patterns of stimulation should be selected for therapeutic advantage in treating various diseases. In this study, we find that even rearranging the order of the same set of varying IPI can change the effect of stimulation pulses substantially. Notably, the IPIs need to vary only in a small range such as 5–10 ms (i.e., 200–100 Hz) with a mean pulse frequency of ∼130 Hz, a commonly used frequency in DBS therapy. In addition, the changes in the orders of varying IPI did not significantly change the total amount of neuronal firing during HFS (Table 3) but only redistributed the firing timing of individual neurons.

Furthermore, our investigation of the neuronal responses to specific IPI episodes in a random IPI sequence provides clues to designing novel temporal patterns of stimulation. The salient changes in the ability of a pulse sequence to recruit a population of neurons by the specific IPI episodes (several shorter IPIs followed by a longer IPI as in Figure 6C) may exert stronger effects on neurons than regular and mild impulses from stimulation with constant IPI. This suggests that adjusting the amount of such IPI episodes may quantitatively regulate the stimulation effect, which acts as a “dose” of stimulation. The recruitment of a large population of neurons may be beneficial for treating diseases caused by excessive suppression of neuronal activity, such as disorders of consciousness after traumatic brain injury. In another perspective, the enhanced effects by random IPI suggest a potential to lower the electrical energy consumption, because only a decreased strength of stimulation may be needed to obtain a similar level of effect as constant IPI. Therefore, the present study with random IPI may inform the design of novel temporal patterns of stimulation that provide therapeutic advantage, less energy consumption, and more safety over regular stimulation for extending DBS applications in treating more brain diseases.

### Limitations of the Study

In the study, the effects of varying IPI were generated from the direct action of stimulation on the neuronal membrane, not involving specific synaptic transmission or neuronal network. Because the mechanisms of action involving the non-linear dynamics of ion channels are universal to the membrane of various neurons in brain, the effects of varying IPI may also apply to neurons in other brain regions directly activated by stimulations. However, synaptic and network involvements may complicate these effects and impose additional modulation to projection neurons in orthodromic propagations, which needs further investigations. Furthermore, even for the antidromic situation, in addition to the depolarization block of axons investigated here, sustained HFS might induce changes in other features along the axon to soma. Also, the stochastic network activity received by soma and the complexity of axonal structures might affect soma’s responses to the axonal HFS. Therefore, more accurate modeling with considerations of these factors is needed to further confirm the results of the present study.

In addition, the rat experiments were performed under anesthesia. The anesthetic urethane could reduce the synaptic responses, elevate the threshold of action potential firing, and decrease the firing rate of neurons (Mercer et al., 1978; Shirasaka and Wasterlain, 1995). Although the antidromic activation paradigm used in the present study did not involve synaptic transmissions, the excitability of soma may be changed by the anesthetic, thereby possibly affecting the responses of neurons to the stimulation. Therefore, further studies are needed to duplicate the results in awake animals.

## Conclusion

The present study suggests that with the same amount of pulses together with the same distribution of IPI, only changing the order of varying IPI can introduce substantial changes in neuronal responses to HFS. Non-linear recovery course of membrane excitability might be the underlying cellular mechanisms resulting in the differences in neuronal responses. The finding provides a new strategy for developing novel stimulation paradigms to modulate the firing patterns of neuron populations, which may improve the DBS efficacy and extend the application of DBS therapy to more brain diseases.

## Data Availability Statement

The original contributions presented in the study are included in the article, further inquiries can be directed to the corresponding author.

## Ethics Statement

The animal study was reviewed and approved by the Institutional Animal Care and Ethics Committee, Zhejiang University, Hangzhou, China.

## Author Contributions

ZF and LZ designed the study. HH and ZW performed the animal experiments. LZ, HH, and YY analyzed the experimental data. LZ created the computational model, and XW contributed to the model modification. LZ performed the simulations. ZF, LZ, and XW interpreted the results and wrote the manuscript. All authors approved the final version for submission.

## Conflict of Interest

The authors declare that the research was conducted in the absence of any commercial or financial relationships that could be construed as a potential conflict of interest.
